# Psychological therapists’ perceptions of adolescent depression and its treatment: A mixed methods online survey

**DOI:** 10.1177/13591045221104570

**Published:** 2022-05-29

**Authors:** Bethany Cliffe, Amelia Peck, Jawairya Shafique, Emily Hards, Maria E Loades

**Affiliations:** 1 Department of Health, 1555 University of Bath, Bath, UK; 2 Department of Psychology, 1555 University of Bath, Bath, UK

**Keywords:** Depression, therapist perceptions, evidence-based therapy, cognitive behavioural therapy, adolescent

## Abstract

**Background:**

Challenges to implementating interventions for adolescent depression exist. Exploring the perceptions of key stakeholders in the treatment of adolescent depression is essential for improving implementation . This study aimed to explore psychological therapists’ perceptions of, and experiences treating, adolescent depression to identify future avenues for exploration.

**Method:**

Data were collected opportunistically via a survey integrated within an e-learning package about adolescent depression.

**Results:**

Participants believed that adolescent depression was characterised by adolescents’ lack of understanding, isolation, and a lack of hope and knowledge. Participants overcame engagement barriers by building trust. Following the e-learning, participants expressed increased understanding of the risk factors associated with adolescent depression and of assessment using different measures. Several key areas for future research to explore were identified and discussed, including (1) whether clinicians of different modalities or at different career stages have difference perceptions, (2) how to meaningfully engage adolescents in treatment and (3) how to train clinicians on different modalities so patients have a choice over their treatment.

**Conclusion:**

This study demonstrates the value of knowledge gained from understanding psychological therapists’ perceptions and illustrates how this can contribute to the improved treatment of adolescent depression.

## Introduction

Depression in young people is prevalent; 1.5% of children and 4.8% of adolescents suffer depressive episodes (NHS Digital, 2018). The NICE guidelines recommend several therapies for adolescent depression, including cognitive behavioural therapy (CBT), interpersonal psychotherapy (IPT), brief psychosocial therapy (BPI), short-term psychodynamic psychotherapy (STPP), and family therapy (FT) (NICE, 2019). Meta-analyses have consistently shown that psychotherapies have moderate effects on adolescent depression (Eckshtain et al., 2020; Cuijpers et al., 2021; Weersing et al., 2017). Additionally, IMPACT, a large randomized control trial (RCT) found that CBT, BPI and STPP were all equally moderately effective in reducing adolescent depression symptoms at a one-year follow-up (Goodyer et al., 2011, 2017; Britten, 2010; Nelson et al., 2006).

### The gap between research and practice

Effect sizes for interventions are often smaller in clinical settings compared to trial settings (Wergeland et al., 2021; Bear et al., 2020). There are several reasons why it may be harder for therapists to implement Evidence-Based Treatments (EBT) in clinical settings, including the complexity of clinical cases; depression is heterogenous with different symptom patterns and co-morbidity is common. One study found that only 42% of adolescents referred to mental health services for depression received a depressive disorder as their primary diagnosis, with 23% meeting the criteria for a primary anxiety disorder and 25% having one or more comorbid diagnoses (Orchard et al., 2017). Comorbidity makes it hard for therapists to implement EBTs (Hetrick et al., 2011) and RCTs often exclude comorbid cases, leaving therapists uncertain about how research applies to clinical practice.

Secondly, adolescent drop-out/disengagement from therapy is common (O’Keeffe et al., 2019), for example, over 33% of adolescents dropped-out of treatment in the IMPACT RCT (O’Keeffe et al., 2018). Compliance with psychological therapy tasks that can predict clinical improvement, such as homework (Simons et al., 2012), is low among depressed adolescents (Gaynor et al., 2006). Research has explored therapist behaviours that promote adolescent engagement, such as understanding adolescents’ experiences and personalizing therapeutic tasks, (Jungbluth & Shirk, 2009; Karver et al., 2008), but not how psychological therapists engage depressed adolescents in clinical practice.

### Improving implementation

Exploring psychological therapists’ perceptions of adolescent depression is essential to improve its treatment; research has shown that misunderstanding depression limits professionals’ abilities to treat it (Britten, 2010; Iliffe et al., 2008; Shafran et al., 2009). Despite this, little research has investigated psychological therapists’ experiences of using EBTs for adolescent depression. Instead, research has prioritised competencies required to deliver interventions and therapists’ adherence to treatment models (Midgley et al., 2018; Sburlati et al., 2012). Additionally, studies have also investigated practitioners’ experiences of implementing EBTs for adolescent depression but have not specifically focused on psychological therapists (Hetrick et al., 2011). Research exploring the perspective of psychological therapists is therefore warranted.

### E-learning as a tool for knowledge

E-learning uses digital resources (Kumar et al., 2018) for educational purposes and is both convenient and widely accessible (Kala et al., 2010). Psychological therapists have asked for additional learning to increase their knowledge and confidence regarding adolescent depression (Jelinek et al., 2013; Hinrichs et al., 2012; Pfefferle, 2007). Research has shown that mental health professionals' knowledge and confidence increased significantly more than a control group after completing an e-learning package on adolescent depression (Ghoncheh et al., 2016). However, no studies have explored the impact of e-learning packages on psychological therapists specifically. Further, the few e-learning programmes created for adolescent mental health have a specific focus, e.g. communication skills (Tchernegovski et al., 2015) or CBT (Westbrook et al., 2012). Consequently, an e-learning package was created to provide psychological therapists with up-to-date information on adolescent depression, assessment, formulation, and multiple EBT’s.

### The present study

We aimed to use this opportunistically collected data to inform potential future research directions for therapist training in and provision of treatments for adolescent depression.

Objective 1: To explore psychological therapists’ perceptions of adolescent depression (e.g. how they perceive the adolescent experience of depression) and their experiences working therapeutically with adolescent depression (e.g. engaging adolescents in treatment, barriers and facilitators of treatment, perceptions of treatments used).

Objective 2: To explore the experiences of psychological therapists completing an adolescent depression e-learning package.

## Method

### Study design

This exploratory study used an online survey with both free-text and Likert style questions. Questions were primarily qualitative, with supplementary quantitative questions also.

### Participants

Individuals providing or supporting psychological therapy who wished to complete an e-learning package on adolescent depression (in English) were eligible to participate.

The final sample consisted of 27 participants who were predominantly female (89%) and white (85%) (see Table 1).

**Table 1.** Participants’ demographic and professional background information (*N* = 27).

Demographics	Group	Frequency (%)
Gender	Female	24 (88.89)
	Male	3 (11.11)
Age	<30	10 (37.04)
	31-40	14 (51.85)
	41+	3 (11.11%)
Ethnicity	White/White British	24 (88.89)
	Asian/Asian British	2 (7.41)
	Other	1 (3.70)
Location	United Kingdom	21 (77.78)
	Germany	1 (3.70)
	Ireland	1 (3.70)
	South Africa	2 (7.40)
	Isle of Man	2 (7.40)
Service setting	NHS health trust	15 (55.56)
	Private practice	1 (3.70)
	Education sector	2 (7.40)
	Voluntary sector	3 (11.11)
	Other	6 (22.22)
Professional background	Clinical/Counselling psychologist	9 (33.33)
	Allied health professional	2 (7.40)
	Counsellor	2 (7.40)
	Trainee/Assistant psychologist	7 (25.92)
	Wellbeing/Educational mental health practitioner	2 (7.40)
	Other	5 (18.52)
Years of experience	<1	7 (25.92)
	1-5	12 (44.44)
	6+	8 (29.63)

### Materials

Demographic questions were completed first, and questions relating to psychological therapists’ perceptions of adolescent depression were embedded throughout (please see supplementary material). The package covered facts about adolescent depression and provided guidance on assessment, formulation, and EBTs including CBT, IPT, BPI, FT and STPP. The package was created by ML, an experienced Clinical Psychologist and Fellow of the Higher Education Academy, with a specialism in adolescent depression. It was developed with input from patient and public involvement stakeholders, including a young person with depression. The content included written information and videos. For example, a video produced as part of the IMPACT-ME study gave information about depressed adolescents’ experiences (Dunn et al., 2018). The package took 2-3 hours to complete, and participants could pause and return later. They could download a PDF of the package and a certificate of completion.

### Qualitative items

Individuals were asked to provide their opinions on adolescent depression, including how they perceived the experience of adolescent depression, barriers that adolescents face in accessing support for depression, how they engage adolescents in treatment, challenges they face in treating adolescents with depression and how they overcome these.

Please see the supplementary material for a list of all items.

### Quantitative items

All items were developed by the researchers to address specific questions and to be as brief as possible. They were asked to rate their confidence in using specific treatment approaches on a scale from 0 (not at all confident) to 4 (very confident). Finally, they were asked to rate how important they perceive each symptom of depression to be on a scale of 0 (not important at all) to 100 (very important).

### Ethical considerations

Ethical approval was granted by the Psychological Ethics Committee of the University of Bath (PREC code 21-003).

### Procedure

Recruitment occurred between February-July 2021. Study adverts were shared via mailing lists including NHS doctorate in clinical psychology practice placement supervisors, on www.drshirleyreynolds.com and via social media pages (Twitter and Facebook). Snowballing was encouraged. Participation was encouraged via the opportunity to receive training in an important area of clinical importance, and a certificate of completing a CPD activity.

Interested participants were informed of the purposes of the study and invited to complete the consent form and continue through the e-learning package. Upon completion, participants were again asked to consent to their responses being submitted. Participants were informed that they could withdraw from the study until they submitted their data without providing a reason. All study documentation was provided via the Qualtrics platform.

### Data analysis

All data analyses were performed by AP and JS, with supervisory oversight from BC and EH.

### Qualitative data analysis

Content analysis followed the steps recommended by Elo and Kyngäs (2008): (1) preparation (becoming immersed in the data); (2) organisation (open coding, generating categories); and (3) reporting the data using the categories (qualitative) or counts (quantitative). A pragmatic approach was taken, assuming knowledge generation to be a never-ending process which can always be revised and improved and applied to real-life scenarios (Feilzer, 2010).

Objective 1: inductive content analysis was chosen due to the lack of previous research, in order to simply report common issues discussed within the data (Elo & Kyngäs, 2008; Hsieh & Shannon, 2005). It is an appropriate analysis method to use within studies that aim to represent a phenomenon from the perspective of the participants (Vaismoradi et al., 2013), even if the study is not philosophically qualitative (Braun & Clarke, 2021). Analysis was conducted on NVivo and considered both the surface and latent meanings (Bush & Amechi, 2020).

Objective 2: content analysis was used to summarise what participants found useful about the e-learning package (Krippendorff, 2004). Content analysis was appropriate here as it allows for the quantification of data, as opposed to thematic analysis, for example (Vaismoradi et al., 2013), which allowed frequency of codes to be presented here to demonstrate overall perceptions of the e-learning package.

### Quantitative data analysis

Quantitative data were analysed by AP and JS using IBM SPSS version 26.

Descriptive statistics were used to summarise responses.

## Results

Objective ***1: To explore psychological therapists’ perceptions of adolescent depression and their experiences working therapeutically with adolescent depression***

Two categories were developed, (1) psychological therapists’ perceptions of adolescent depression, and (2) psychological therapists’ experiences of working therapeutically with adolescent depression:

### Psychological therapists’ perceptions of adolescent depression

All participants responded to the questions that informed the development of this category. Analysis produced four subcategories:

#### Adolescents’ lack of understanding

Participants believed adolescents did not understand their symptoms, often using words like ‘confusing’ or ‘scary’ to describe adolescents’ experiences. They suggested that adolescents may not recognise their symptoms as depression. For example, they referred to adolescents struggling to “understand what’s happening to them” (trainee/AP, 1-5 years’ experience) or confusing their symptoms with “normal teenage stuff” (counsellor, 1-5 years’ experience). Participants also believed that adolescents lacked understanding about how to seek support, including “not knowing who to speak to” (clinical/counselling psychologist, 6-10 years’ experience) and “not knowing what to expect” (trainee/assistant psychologist (AP), <1 year experience).

#### Other peoples’ lack of knowledge about depression

Participants believed that adolescents’ “family” and “friends” lacked knowledge and understanding about depression. For example, participants discussed “people not understanding what depression is” (psychotherapist, 6-10 years’ experience) or holding “stigma” about it. Participants suggested that this lack of knowledge may result in adolescents’ struggle being undermined, including “parents not believing that depression exists” (mental health/wellbeing practitioner, 1-5 years’ experience). Finally, participants suggested that those around the adolescent had a “lack of awareness of mental health services and pathway[s]” (research psychologist, <1 year experience), which acted as a direct barrier to adolescents seeking support.

#### Depression as an isolating experience

Participants used words like “lonely” and “alienating” to portray adolescent depression as an isolating experience. This was firstly due to adolescents not communicating either because they experienced “difficulties showing people what’s happening” (clinical/counselling psychologist, 6-10 years’ experience) and not being “open and honest about their feelings” (psychotherapist, 6-10 years’ experience). Secondly, participants noted a “lack of accessible support” (trainee/AP, 1-5 years’ experience) which meant adolescents “feel like no one else can see or understand them” (trainee/AP, <1 year experience) and that they do “not have the right people to speak to” (trainee/AP, <1 year experience).

#### Depression as a dark place

Participants discussed adolescents “feeling hopeless about the future” (clinical/counselling psychologist, 6-10 years’ experience) and “not feel motivated to seek help” (clinical/counselling psychologist, 6-10 years’ experience), portraying depression as a dark and hopeless experience. Participants also highlighted the serious risks associated with adolescent depression by using words like “debilitating” and “suicidality”.

### Quantitative

**Figure 1.**
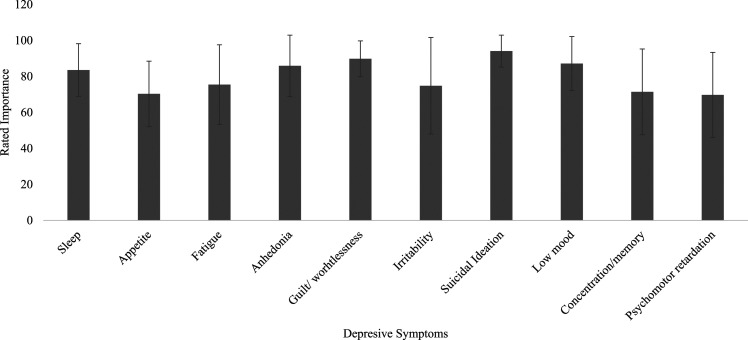
The rated importance of depressive symptoms.

As seen in Figure 1, participants rated suicidal ideation as the most important symptom to address (*M* = 94.09, *SD* = 8.90), although there were no significant differences between any of the symptoms rated.

### Psychological therapists’ experiences of working therapeutically with adolescent depression

Twenty-five participants responded to some/all of the questions that informed this category. Analysis generated five subcategories:

#### Engagement of the adolescent and their family as challenging

“Engagement” of the adolescent, including “not attending sessions” (clinical/counselling psychologist, 6-10 years’ experience) was discussed as a challenge to delivering EBTs. Participants also emphasised the importance of family engagement. For example, “parents struggling to support home practice” (clinical/counselling psychologist, 1-5 years’ experience) was presented as a challenge and “involving family members” (clinical/counselling psychologist, 1-5 years’ experience) was presented as a solution to adolescents’ engagement issues. However, participants discussed “making sure the young person is on board” (trainee/AP, 1-5 years’ experience) before engaging the family, due to adolescents’ resistance against family involvement.

#### Building trust

Building trust with the adolescent was central to participants’ experiences. For example, “building a therapeutic relationship” (counsellor, 1-5 years’ experience) was used to increase adolescents’ engagement. Participants also highlighted the importance of creating “a safe space for opening up without shame or judgement” (trainee/AP, 1-5 years’ experience). The role of listening and understanding was important for building trust and engaging adolescents, as participants discussed using “compassion” and “buckets of empathy and listening to engage” (clinical/counselling psychologist, 1-5 years’ experience).

#### Empowering the adolescent

Participants’ experiences of working therapeutically with depressed adolescents involved making “them feel autonomous and empowered” (trainee/AP, 1-5 years’ experience) by giving them choice, control and increasing their hope for the future. For example, participants discussed taking a “collaborative approach” (clinical/counselling psychologist, 1-5 years’ experience) with the adolescent including “agreeing plans for sessions” and “let[ting] them lead the discussion” (mental health/wellbeing practitioner, 1-5 years’ experience). Participants also highlighted the importance of “instil[ling] hope” (trainee/AP, <1 year experience) for the future, to empower young people to engage.

#### Treating each adolescent as an individual

Participants’ experiences involved treating each adolescent as an individual, either by adapting to their needs or by understanding them as individuals. For example, participants discussed the importance of “get[ting] to know their story” (trainee/AP, 1-5 years’ experience) and “understanding their contexts” (clinical/counselling psychologist, 1-5 years’ experience) to make therapy “meaningful” and “relevant to the individual” (teaching assistant, <1 year experience. This was also used to increase engagement, for example: “I endeavour to creatively use their particular hobbies/interests and preferences to deliver CBT in an engaging way for each young person” (trainee/AP, 1-5 years’ experience).

#### The importance of resources

Participants discussed the importance of resources such as “supervision” and “training” as facilitators for EBTs, while “research” and “guidelines” were cited as facilitators for implementation. Participants emphasised the need for these materials to be “readily available” (counsellor, 6-10 years’ experience), “up to date” (clinical/counselling psychologist, 1-5 years’ experience) and “easy to follow” (trainee/AP, 1-5 years’ experience). Finally, participants cited “time constraints” (trainee/AP, 1-5 years’ experience) as a challenge to EBTs and “time to refer back to materials” (clinical/counselling psychologist, 6-10 years’ experience) as a facilitator. This suggests sufficient time as well other resources can affect psychological therapists’ experiences of implementing EBTs.

Table 2 presents the descriptive statistics for participants’ reported use of EBTs and their confidence in delivering EBTs. CBT was most used by participants, and they reported having the most confidence in delivering CBT. Confidence was rated on a scale from 0 (not at all confident) to 4 (very confident). Most participants only reported using one treatment (11, 45.83%), followed by two treatments (10, 41.67%), three treatments (2, 8.33%) and lastly only one person (4.17%) reported using four treatments.

**Table 2.** Participants’ reported use and confidence for each evidence based interventions (*n* = 24).

Treatment type	Number of participants who use each treatment (%)	Mean confidence (SD)
BPI	8 (33.33)	2.35 (1.27)
CBT	19 (79.17)	3.33 (1.13)
PP	1 (4.17)	1.57 (0.90)
IPT	3 (12.50)	1.92 (1.10)
FT	5 (20.83)	1.96 (0.98)
Other	4 (16.67)	N/A

*Note.* Responses for ‘other’ were: “systemic, narrative or attachment’, “integrative’, “behavioural activation” and “family-based therapy”.

*Note.* One participant reported their confidence for only two interventions (CBT and IPT).

Objective **2:** To explore the experiences of psychological therapists completing an adolescent depression e-learning package.

22 participants responded to the question ‘What has been most useful about this e-learning package?’. The most common codes were grouped into 8 categories (See Table 3). The most valuable aspect of the e-learning package was the variety of resources (*n* = 13, 59%). Participants “loved” that the e-learning package included “a lot of information and that the videos were very helpful” (allied health professional, <1 year experience). Furthermore, the videos on “sleep and CBT were particularly useful” (clinical/counselling psychologist, 1-5 years’ experience) and the links to research articles were something that they would “be able to refer back to” (clinical/counselling psychologist, 6-10 years’ experience) in the future. The e-learning package was useful in strengthening participants’ knowledge about adolescent depression (*n* = 7, 32%). Going through the package was a “helpful refresh of knowledge and skill” (clinical/counselling psychologist, 1-5 years’ experience) and gave participants the “confidence to support young people” (chaplain, <1 year experience) more effectively. It was found to be “advantageous to go over the whole process” (trainee/AP, 1-5 years’ experience) from assessment and formulation to interventions. Participants found it practical to have an overview of various interventions that can be used to treat adolescents with depression (*n* = 6, 27%). They felt the e-learning package provided them with the “opportunity to take a meta-perspective on the different approaches” (clinical/counselling psychologist, 1-5 years’ experience) related to adolescent depression.

**Table 3.** What has been useful to you about this E-learning package? (*n* = 22).

Categories	*n*	%
The range of resources	13	59
Reinforcing their knowledge about adolescent depression	7	32
Overview of various interventions	6	27
Different measures for assessment and formulation	5	23
Linking obtained knowledge to practice	5	23
Opportunity to reflect on their learning	2	9

## Discussion

We sought to use opportunistically collected data to explore how psychological therapists perceive adolescent depression and their experiences of completing an adolescent depression e-learning package, and thus to generate directions for future research. One key finding is that psychological therapists perceived adolescent depression to be a dark, isolating experience poorly understood by adolescents and others. This is consistent with previous research suggesting that adolescents feel isolated, confused, hopeless, and have a negative view of life (Dunn et al., 2018; Midgley et al., 2015). Future research could expand on this finding and thus help to identify specific training needs further by examining whether psychological therapists at different stages of their training or those trained in different modalities of therapy have different perceptions of the experience of adolescent depression.

Another key finding is that psychological therapists rated suicidal ideation as the most important symptom to address during therapy and psychomotor changes as the least important. This reflects findings from Orchard et al. (2017) who identified suicidal ideation in 86% of adolescents diagnosed with a depressive disorder referred to a community mental health service and identified psychomotor changes in only 19% (2017). It is also reassuring, given that suicidal ideation is a key aspect of risk assessment. However, what is important to highlight is that in our study, participants rated all symptoms as important to address (≥60 out of 100), corroborating findings that therapists perceive depression recovery as a holistic process involving multiple symptomatic outcomes (Krause et al., 2020).

Our participants perceived there to be challenges in engaging the adolescent and their family in therapeutic work. This is consistent with findings that adolescents themselves perceive engagement issues within therapy (Gaynor et al., 2006; O’Keeffe et al., 2018) and family involvement in treatment can pose challenges to psychological therapists (Iliffe et al., 2008; Wells & Albano, 2005). To overcome these engagement challenges, participants build trust, empower the adolescent and treat them as individuals. This corroborates research suggesting that adolescents value therapists who encourage autonomy (Wilmots et al., 2020; Wisdom et al., 2006), and points towards the need for more of a research focus into how to engage adolescents in therapy in ways that are meaningful and relevant to them, as well as to better understand the potential barriers to engagement in therapy for this group.

Our participants most commonly used and were most confident in delivering CBT. This is unsurprising as CBT is well-established as a recommended EBT for adolescent depression (Brent et al., 1997; NICE, 2019) and has been identified as the dominant intervention within the literature (Weersing et al., 2017). In clinical practice, CBT has been reported as the most common treatment offered for depression (Clark, 2011) but the NICE guidelines suggest therapists should be able to offer adolescents therapy options before selecting one (NICE, 2019). However, participants here lacked confidence delivering other EBTs suggesting this may not be possible in practice. Future research is needed to examine how best to skill up the workforce to enable services to offer the range of evidence-based options to adolescents with depression and to know how best to enable and empower them to make informed choices based on their preferences.

### Limitations and directions for future research

As the measures were embedded within an extensive e-learning package, they were kept brief to not over-burden participants. However, the brevity of the measures may have been at the expense of detail; future research may benefit from focus groups or interviews with a subset of participants. The extensive nature of the e-learning package also meant that attrition rates were high and the sample size was consequently very small and not sufficiently powered. Quantitative results must therefore be interpreted with caution as they are unable to be generalised outside of this study

Other methodological issues stem from the snowballing recruitment technique used which meant it is hard for the researchers to estimate the proportion of participants who completed this out of the total possible number of participants who were made aware of the study. The time participants spent engaged with the training was also not captured, so the researchers are unaware of the average time taken to complete this package, though it was estimated to be around 2-3 hours. As participants were not required to complete the survey items embedded throughout the package to access the e-learning, it is not possible for the researchers to know exactly how many participants completed the training. Thus it is only possible to know how many participants completed the questions. Future studies would benefit from capturing these missing data.

Finally, there was a lack of diversity among participants. A predominantly white, female sample is not representative of all psychological therapists working with young people. However, this does reflect the current state of the psychology workforce. Research has shown that only 9.6% of all qualified clinical psychologists are Black, Asian and/or Minority Ethnic (BAME) (Office of National Statistics, 2018). Until the state of our workforce reflects the true diversity of society, it may be difficult to get a sample as diverse as our population.

### Clinical implications

These findings highlight a need to increase psychological therapists’ confidence in delivering FT, IPT and PP. Not all adolescents respond to CBT (Stikkelbroek et al., 2020) and it is therefore important that psychological therapists are able to base the treatment option on the patients’ needs and preferences, rather than their own confidence levels (NICE, 2019).

Secondly, these findings reflect a need for psychological therapists’ perspectives to be incorporated into guidelines and protocols for treating adolescent depression, as participants demonstrated valuable knowledge, including ways to increase adolescents’ engagement. To the best of the authors knowledge however, no research has investigated how these methods can be evaluated and incorporated into protocol.

Finally, these results support the use of e-learning as an effective way to keep professionals aware of the latest and effective diagnostic tools and screening instruments for assessment and formulation, as research has shown many psychological therapists are not always aware of these resources (Sheldrick et al., 2011).

## Conclusion

Psychological therapists’ perceived there to be a lack of understanding around adolescent depression, both from the adolescent themselves and those around them. They identified engagement as a challenge to treating adolescent depression but discussed how they attempt to overcome this. Participants mostly used and felt most confident using CBT over and above other recommended treatments, highlighting the need for more extensive training in the range of evidence-based therapies for adolescent depression to ensure that adolescents are offered choices. It was highlighted that future research should explore (1) how clinicians of different modalities or at different stages of their career perceive adolescent depression, (2) how adolescents can be encouraged to engage meaningfully in treatment, and (3) how clinicians can best be skilled up on different treatment modalities to ensure patients have a choice over what treatment they receive.

## Supplemental Material

Supplemental Material - Psychological therapists’ perceptions of adolescent depression and its treatment: A mixed methods online survey Click here for additional data file. Supplemental Material for Psychological therapists’ perceptions of adolescent depression and its treatment: A mixed methods online survey by B Cliffe, A Peck, J Shafique, E Hards and ME Loades in Clinical Child Psychology and Psychiatry
