# Supplementary material for: Psychological therapists’ perceptions of adolescent depression and its treatment: A mixed methods online survey
Source: Clin Child Psychol Psychiatry. 2022 May 29;28(2):580–94. doi: 10.1177/13591045221104570 (PMC10018054; doi:10.1177/13591045221104570)
Supplement: Supplemental Material - Psychological therapists’ perceptions of adolescent depression and its treatment: A mixed methods online survey [file sj-pdf-1-ccp-10.1177_13591045221104570.pdf]

Table 1
Survey questions addressing each aim

Research Aim	Survey Questions
1) Explore psychological therapists' perceptions and experiences of adolescent depression and its treatment	1) What 3 words would you use that best describe the experience of adolescent depression?<br>2) What common barriers/difficulties to accessing help do adolescents with depression tend to report?<br>3) What do you do to engage adolescents with depression in treatment?<br>4) What are the biggest challenges you encounter when working therapeutically with adolescents who have depression?<br>5) What do you do to reduce or overcome these challenges?<br>6) What enables you to do evidence-based practice when working with adolescents who have depression?<br>7) What gets in the way of evidence-based practice when working with adolescents who have depression?

2) Explore the experiences of psychological therapists of completing an adolescent depression e-learning package.	1) What has been most useful to you about this e-learning package?
